# Integrating a model of care within a community-based managed alcohol program with indigenous men experiencing homelessness and alcohol use: a retrospective chart review

**DOI:** 10.1186/s12954-026-01493-z

**Published:** 2026-07-07

**Authors:** Natalia Gala, Manuela Mbacfou Temgoua, Annie Talbot, Jorge Flores-Aranda, Rossio Motta-Ochoa, Matthew Biddle, Mark Alsop, Stéphanie Marsan

**Affiliations:** 1 Centre de recherche du Centre hospitalier de l’Université de Montréal, Montréal, QC Canada; 2 Département de médecine de famille et médecine d’urgence, Université de Montréal, Montréal, QC Canada; 3 École de travail social, Université du Québec à Montréal, Montréal, QC Canada; 4 École de travail social, Université de Montréal, Montréal, QC Canada; 5 Projets Autochtones du Québec, Montréal, QC Canada

**Keywords:** Indigenous health, Managed alcohol program, Harm reduction, Alcohol use disorder, Homelessness, Addiction medicine

## Abstract

**Background:**

In Montréal, many members of the Indigenous community are affected by homelessness and substance use, explained in part by the loss of cultural identity. These challenges are compounded by barriers to health and social services, and reduced shelter capacity during the pandemic. In response, an urban Indigenous-led community organization developed a managed alcohol program (MAP) in partnership with a university hospital (UH) addiction medicine team in Montréal, Canada. We describe health outcomes, service access, and substance use among Indigenous men experiencing homelessness and severe alcohol use enrolled in the MAP.

**Methods:**

A retrospective medical chart review of residents admitted to the MAP was conducted following its opening in December 2020. Descriptive data were abstracted from the university hospital’s electronic medical records and the MAP’s medical charts. Pre-admission outcomes were captured for the one-year period prior to the start of the program (December 2019 to December 2020), and post-admission outcomes were collected during each resident’s observed follow-up period for up to one year after program entry (December 2020 to December 2021). Descriptive analyses were used to summarize resident characteristics and outcomes.

**Results:**

Findings are reported for 12 Indigenous men enrolled in the program during the study period. Residents had a mean age of 51 years, and the majority identified as Inuit (41.7%). The mean duration in the MAP was 208.3 days (range: 32–396 days). Most residents reported an underlying health condition (83.3%), diagnosed psychiatric disorder (75.0%), and substance use disorder other than alcohol (75.0%). During the observed follow-up period, mean alcohol use decreased from 29.1 to 20.0 drinks per day. Decreased use of other substances, including cannabis and tobacco, was also observed. Emergency department (ED) utilization was reportedly lower during follow-up, with 12 residents accessing ED services prior to admission compared to 5 during follow-up.

**Conclusions:**

A partnership between an urban Indigenous-led community organization and an addiction medicine team can support harm reduction and access to care. Findings suggest that similar collaborative models may be relevant for supporting care among Indigenous populations experiencing homelessness and severe alcohol use.

## Background

People experiencing homelessness face disproportionate health burdens and substantial barriers to accessing health and psychosocial services, largely driven by residential instability and social precarity [1]. In Montréal (Tiohtià:ke), a 2022 point-in-time count conducted by the Ministry of Health and Social Services identified 4,690 individuals experiencing visible homelessness, defined as lacking a permanent and safe residence and staying in places not meant for human habitation or in temporary shelters. Indigenous Peoples were disproportionately affected, representing 13.2% of this population despite accounting for only 1.1% of the general population [2, 3].

The enduring impacts of colonization have contributed to profound inequities in health and social outcomes among Indigenous Peoples. Structural factors such as poverty, systemic racism, and substance use (particularly alcohol use) further limit access to appropriate and timely care [4]. Experiences of stigma and discrimination within healthcare settings further exacerbate medical mistrust and frequently lead to delayed or avoided care [5–8]. Moreover, sobriety requirements imposed by many traditional health and shelter services often exclude individuals with severe alcohol use disorder (AUD), further limiting access to essential supports [9].

In response to these intersecting challenges, a culturally safe, partnership-based model of care was established through a managed alcohol program (MAP). Rooted in harm reduction principles, the MAP aimed to promote security, stability, and peace of mind, while providing distinctions-based Indigenous cultural supports alongside regulated alcohol provision, supportive housing, and health services consistent with established MAP models [10, 11]. Existing evidence suggests that MAPs are associated with reductions in alcohol-related harms, hospital admissions, and emergency visits, while improving housing stability, access to healthcare, and overall well-being [10, 12–15]. Although MAPs are increasingly implemented across Canada, the majority are community-based, with relatively few formally linked to medical institutions [16]. To our knowledge, no MAP has previously described integration between a university hospital addiction medicine team and an urban Indigenous-led community organization in Canada to support on-site care and facilitate access to specialized health services. The current study performed a retrospective medical chart review to describe health outcomes and healthcare access among Indigenous men experiencing homelessness and alcohol use disorder enrolled in this integrated model of care.

## Methods

### Program description

#### Residential setting

The MAP was implemented in December 2020, during the COVID-19 pandemic, by an urban Indigenous-led community organization (hereafter referred to as the Community Organization [CO]) in Montréal as a pilot initiative developed in partnership with a University Hospital’s (UH) addiction medicine service. At the time of implementation, it was the first residential MAP in Québec. The CO supports urban-based First Nations, Inuit, and Métis (FNIM) individuals experiencing or at risk of homelessness and provides services such as transitional and permanent housing, in addition to a shelter, grounded in Indigenous values and traditions. At the time of evaluation, the MAP was housed in a rented residential facility. Since then, it has relocated to a residential building owned and operated by the CO, in proximity to the UH. The garden-level floor houses the eight-resident MAP, while the upper levels offering permanent housing, defined as a long-term, stable living arrangement for Indigenous individuals not enrolled in the MAP facing housing insecurity.

The program was initially designed for FNIM men experiencing homelessness, significant physical and mental health vulnerabilities, and AUD. It provided supportive housing intended to serve as the residents’ long-term home, alongside managed alcohol provision, access to primary healthcare services, and support for residents in connecting or reconnecting with their Indigenous culture.

Each resident had a private room within the MAP, with access to shared spaces including a kitchen, dining area, and a reception area where alcohol was dispensed. Residents made a monthly financial contribution, adjusted to their income, to partially cover housing and alcohol costs and were free to come and go from the residence. The program provided 24/7 support, including psychosocial, community health, and life-skills services, as well as cultural programming. Care was delivered within a trauma-informed, harm reduction–oriented framework, emphasizing non-judgmental engagement and meeting residents where they were to support stabilization and safety.

#### Security

To support the safety of both residents and staff, clear criteria were established to guide discharge and suspension decisions. Voluntary discharge occurred when a resident chose to leave the program, whereas involuntary discharge was determined by the interdisciplinary team when a resident was no longer considered appropriate for the program. Reasons for involuntary discharge included persistent unsafe behaviour (e.g., threats or hate speech), repeated non-adherence to program agreements, or care needs exceeding the program’s capacity. Immediate discharge was reserved for situations involving elevated risk, such as physical violence, while other cases followed a progressive three-strike process. Involuntary suspensions were used only in response to acute safety concerns, including verbal threats of aggression, and ranged from 2 to 30 days. MAP staff employed a trauma-informed approach and exhausted all possible interventions before resorting to suspension or expulsion. Whenever possible, staff focused on supporting residents to remain in the program through conflict resolution and individualized supports. During suspension periods, residents were temporarily relocated to the CO’s shelter or, when feasible, stayed with family or friends.

#### Cultural safety

Cultural safety was a foundational component of the MAP and was reflected in care approaches that respected Indigenous values, practices, and experiences, including the impacts of trauma and colonization. This was achieved through flexible, individualized care rather than standardized protocols, recognizing the diversity of resident’s cultural identities. Cultural practices were incorporated based on individuals’ preferences and included ceremonies, crafts (e.g., stone carving), music, gatherings, access to traditional foods, and the use of non-Western medicines (e.g., smudging and traditional teas). Weekly visits from an Indigenous Elder further supported culturally grounded counseling and care. Cultural safety also involved non-judgmental engagement that acknowledged diverse health beliefs and supported residents in maintaining or restoring connections to their culture and community. Efforts were made to recruit staff who were open to learning from the community and who possessed foundational knowledge on Indigenous realities.

A core component of the program was fostering a sense of community within the residence, while also recognizing residents’ connections to broader communities in Montréal and their home communities. Supporting these relationships and reducing associated social harms were central to the program’s harm reduction approach. In parallel, the integration of the addiction medicine team facilitated the incorporation of cultural elements within the hospital setting, including weekly beading activities, and visits from an Indigenous healer, an addiction counselor and community navigators. Overall, CO staff and UH medical team members took the time to understand and implement culturally safe practices by respecting and learning from residents’ cultural worldviews, values, and practices.

#### Integrated care team

MAP staff included a program coordinator, one daytime and one nighttime intervention worker, a caseworker, a part-time integrated nurse and an Indigenous Elder. Intervention workers were available 24/7; however, due to increasing safety concerns among female intervention workers, a security guard was later added during the evening hours to support staff safety. The UH addiction medicine team consisted of a physician and clinical nurse who conducted weekly on-site visits during the first year, with the physician available 24/7 for emergencies. Primary and addiction care were provided on-site, while specialist care (e.g., gastroenterology) was accessed through referrals to the UH and other healthcare settings. The integrated nurse added to the MAP worked closely with the medical team to support ongoing care.

The UH team collaborated with the CO to provide training on substance use disorders (SUDs), intoxication, overdose, and withdrawal management, substance-induced psychosis, and medication dispensing. They were also involved in the development of program eligibility criteria, policies, procedures, and clinical protocols. In addition, the UH team developed and implemented alcohol dispensing protocols and conducted medical evaluations of potential participants. This collaboration was particularly important given the proximity of services and the need for on-site access to medical care. Through routine visits, the medical team supported engagement with health services and facilitated access to care.

Prior to admission, each resident is evaluated and received a full medical assessment by the UH team at the hospital. The UH addiction physician worked with the resident and, based on the amount of alcohol consumed prior to admission, developed an individualized alcohol plan, which the resident agreed on and which was shared with the MAP team. To support this plan, intervention workers dispensed an average of one 355-ml can of beer (6% alcohol) every one to two 2 h between 7:00 am to 11:00 pm, with the possibility of residents receiving an additional one to two more doses during the night.

Each resident was provided with adequate amounts of alcohol to prevent withdrawal symptoms. Consumption of outside alcohol was prohibited within the program. If a resident was considered overly intoxicated by the organization’s staff, in accordance with evidence-based protocols, an alcohol serving could be refused. Episodes of intoxication were assessed using an intoxication assessment tool based on four domains: speech, coordination, mental status, and level of consciousness. Each domain was categorized across four levels of severity (A–D), with A indicating minimal impairment and D, indicating the highest level of impairment.

#### COVID-19 pandemic

The MAP was implemented during the Coronavirus Disease 2019 (COVID-19) pandemic amid public health restrictions in Quebec that significantly reduced access to shelter and healthcare services for individuals experiencing homelessness and substance use in Montréal. COVID-19 mitigation measures, including mask use and regular testing, were introduced at program initiation, although adherence to mask use varied among residents. As the study relied on retrospective chart abstraction, systematic assessment of COVID-19-related outcomes was not conducted, aside from documentation of COVID-19-related hospitalizations.

### Sample

During the first year of the MAP, Indigenous men were referred by the CO and affiliated community resources. Participants had been living in Montréal and had accessing services through the CO prior to enrollment. Program candidates were identified and evaluated by the CO staff in collaboration with the UH medical team based on predefined inclusion and exclusion criteria. Inclusion criteria were: (1) self-identification as an Indigenous male; (2) age between 18 to 75 years; (3) current experience of homelessness; (4) severe alcohol use disorder (AUD), defined as meeting six or more DSM-V diagnostic criteria [17]; (5) being in the precontemplation or contemplation stage of change according to the Transtheoretical Model [18]; (6) heightened vulnerability to risks associated with living on the street, including exposure to assault or the presence of severe physical or mental health comorbidities; and (7) voluntary participation in the program. Exclusion criteria included: (1) a documented history of aggressive or violent behavior; or (2) the presence of a severe substance use disorder involving substances other than alcohol. All eligible participants who entered the program during the first year of operation were included in the chart review. Thirteen Indigenous men entered the program during the study period; however, one enrolled near the end of data collection and was not included. Therefore, descriptive findings are reported for the remaining 12 residents.

### Outcomes and analysis

A retrospective chart review was conducted using descriptive analyses of data abstracted from medical charts. These findings complement previously published qualitative findings on the MAP [19]. Variables were primarily binary and indicated whether a given outcome was documented during the specified observation period (yes/no), such as the occurrence of an emergency department (ED) visit for any health-related reason. Outcomes were extracted from the UH’s electronic health records and the MAP’s internal documentation, which included both paper-based medical charts and entries stored on the program’s electronic platform (Bitrix). All chart data were entered into REDCap by the research team and analyzed using descriptive statistical methods.

All 12 residents’ medical charts were reviewed for the one-year period prior to the start of the MAP (December 2019–December 2020) to capture healthcare utilization and documented clinical outcomes before program participation. Baseline demographic and health characteristics were obtained from medical assessments completed at program entry for all residents prior to enrolment in the MAP. Outcomes were then assessed during the one-year follow-up period after MAP entry (December 2020–December 2021) for all residents enrolled in the program, regardless of duration or variability of participation. Outcomes were summarized as proportions of residents with at least one documented occurrence during the observation period. Continuous variables, including duration of program participation and length of suspension, were summarized using means and ranges. Alcohol use during the program was measured as daily consumption (drinks per day) and averaged across all residents who entered the program, based on chart-documented intake.

Daily alcohol consumption (e.g., number of standard drinks per day) was calculated using the Canadian Institute for Substance Use Research’s (CISUR) alcohol calculator, which standardizes alcohol volume into equivalent drink units [20]. Healthcare utilization data were extracted from UH records and included ED visits, hospital admissions, and prescription medications.

Informed consent and approvals from the Research Ethics Board (REB) and participating institutions were obtained, permitting access to residents’ medical records. The study protocol and chart review procedures were developed and approved in consultation with Indigenous members of the CO’s Board of Directors. The study was conducted in accordance with the *Tri-Council Policy Statement: Ethical Conduct for Research Involving Humans (TCPS)*, Chapter 9 [21], with the CO engaged throughout all phases of the research.

## Results

Residents had a mean age of 51 years (Table 1). Four participants identified as First Nations, representing one or more of the following Nations: Abenaki, Algonquin, Atikamekw, Cree, Innu, Maliseet, Mi’kmaq, or Naskapi. The majority of residents identified as Inuit, while three residents identified as belonging to another First Nations group or did not report their Indigenous affiliation.

**Table 1:** Participant characteristics at program entry (N = 12 residents)

Characteristics	No. (%) of residents	Mean (Range)
Sex, male	12 (100%)	
Age (years)		51 (34–67)
Indigenous identity		
First nations^1^	4 (33.3%)	
Inuit	5 (41.7%)	
Other or not reported	3 (25.0%)	
Program measures		
Length of time in map (days)		208.33 (32–396)
Length of time in MAP among deceased residents (days)		257.33 (209–335)
Length of suspension (days)		34 (0–162)
Medical history		
Sexually transmitted and blood-borne infections	4 (33.3%)	
(HIV, Hepatitis C)		
Anemia	3 (25.0%)	
Head trauma	4 (33.3%)	
Other health conditions^2^	10 (83.3%)	
Psychiatric history		
Diagnosed psychiatric disorder	9 (75.0%)	
(Anxiety, depression, PTSD)		
Substance use disorder	9 (75.0%)	
(Benzodiazepines, cannabis, cocaine/crack, heroin, other opioids)		

^1^First Nations includes one of Abenaki, Algonquin, Atikamekw, Cree, Innu, Maliseet, Mi’kmaq or Naskapi

^2^Other Health Conditions included Diabetes, Hypertension, Cirrhosis, Chronic Pain, Heart Failure, Cardiac Arrhythmia, Mallory-Weiss tear, Epilepsy, Fatty Liver disease, and Erosive Gastritis

### Program participation and retention

During the first year of implementation, the program experienced ongoing participant turnover due to death (n = 3), suspension (n = 5), and involuntary discharge (n = 4). The mean duration of suspension among residents was 34 days, with suspensions occurring in response to safety-related concerns. Involuntary discharge was documented in cases involving aggression or behavioral issues that exceeded the program’s capacity to ensure safety. Including all residents (those who died, were suspended, or were discharged), the average duration of residency in the MAP was 208.3 days (Table 1). Residents who died during follow-up had among the longest durations of residency within the program (257.33 days).

### Health characteristics at program entry

Comprehensive health assessments conducted by the UH team at program entry documented residents’ medical histories across multiple domains. All residents met diagnostic criteria for severe AUD. Histories of other SUDs (e.g., opioids, benzodiazepines, cannabis), were commonly documented, and most of the residents also had a diagnosed psychiatric disorder and at least one underlying chronic medical condition (Table 1).

### Healthcare utilization and clinical outcomes

All residents had at least one documented ED visit at the UH prior to MAP admission (Table 2). During the follow-up period, five residents accessed ED services. No ED visits related to alcohol withdrawal or overdose were documented during follow-up. The proportion of residents hospitalized at least once remained relatively stable from pre-admission period to follow-up (Table 2).

**Table 2:** UH service use and health characteristics at admission and follow-up in the managed alcohol program (MAP) (N = 12 residents)

	Pre-admission	One YEAR FOLLOW-UP
Characteristic		
Visited emergency department	12 (100%)	5 (41.7%)
Episode of intoxication	9 (75.0%)	4 (33.3%)
Withdrawal (alcohol)	5 (41.7%)	0 (0.0%)
Overdose	2 (16.7%)	0 (0.0%)
Other^1^	12 (100%)	5 (41.7%)
Residents hospitalized	6 (50.0%)	5 (41.7%)
Episode of intoxication	0 (0.0%)	1 (8.3%)
Withdrawal (alcohol)	4 (33.3%)	3 (25.0%)
Overdose	0 (0.0%)	0 (0.0%)
Other^2^	4 (33.3%)	5 (41.7%)
Prescription medication^3^	9 (75.0%)	11 (91.7%)
Experience of harmful consequences linked to alcohol and/or psychoactive substances	11 (100%) (undocumented: 1)	2 (66.7%) (undocumented: 9)

^1^Other reasons for ED visits included fracture, decompensated heart failure, psychosocial difficulties, convulsions, trauma (including head trauma and thoracic trauma), pain (including low back pain, epigastric pain, abdominal pain, chest pain), suicidal thoughts, request for hepatitis C treatment, COVID-19 positive status, acute exacerbations of chronic obstructive pulmonary disease (AECOPD), aggression, and contusion

^2^Other reasons for resident hospitalizations included anemia, pain (fracture), heart failure, cirrhosis, alcoholic hepatitis, cellulitis, abscess, COVID-19 infection, pneumonia, and withdrawal seizures

Resident’s clinical profiles were further characterized through prescription medication use. Medications prescribed included antipsychotics (e.g., risperidone, quetiapine) and treatments for chronic respiratory conditions (e.g., inhalers for asthma or COPD). Relapse prevention medications (e.g., naltrexone, acamprosate) and vitamin supplementation were prescribed at program initiation by the UH addiction physician; however, residents declined these medications. Following MAP admission, a greater proportion of residents received prescription medications for chronic medical conditions through the UH medical team (Table 2). Documentation of substance-related harms (e.g., altercations, alcohol withdrawal, overdose) prior to program admission suggested a high burden of risk; however, documentation during follow-up was incomplete. For nine residents, no recorded information on substance-related harms was available during the follow-up period, limiting the ability to draw conclusions regarding changes in these outcomes.

### Follow-up care

All twelve residents had at least one scheduled follow-up appointment with UH-affiliated healthcare providers during the follow-up period. Follow-up care was provided by the addiction medicine physician, specialist physicians, community nurse practitioners, and other allied health professionals (Table 3).

**Table 3:** Follow-up care with UH-affiliated health specialists (N = 12 residents)

Specialist	n (%) Referred
Family doctor	0 (0.0%)
MAP doctor (UH)	6 (50.0%)
Medical specialist (Gastroenterologist, Hepatologist, Cardiologist, Microbiologist)	7 (58.3%)
Nurse practitioner	11 (91.7%)
Dentist	0 (0.0%)
Other health professional (podiatrists, chiropractors, or physiotherapists)	6 (50.0%)

### Substance use patterns

Self-reported substance use patterns are presented in Table 4. Mean reported alcohol consumption decreased from 29.1 standard drinks per day prior to MAP admission to 20.0 drinks per day during follow-up. During follow-up, fewer residents reported use across most substance categories. Use of gamma-hydroxybutyrate (GHB) use was newly reported by one resident.

**Table 4:** Alcohol Use and self-reported substance use at admission and during follow-up in MAP (N = 12 residents)

Substance use	At admission	One year follow-up
Alcohol use, mean (range)	29.1 (9.0–55.8)	20.0 (9.0–39.5)
Other substances, n (%)		
Opioids	2 (16.7%)	0 (0.0%)
Stimulants	4 (33.3%)	2 (16.7%)
Hallucinogens	1 (8.3%)	1 (8.3%)
Cannabis	12 (100%)	9 (75.0%)
Tobacco	9 (75.0%)	3 (25.0%)
GHB	0 (0.0%)	1 (8.3%)

Values are presented as mean (range) number of standard drinks per day for alcohol use, and n (%) for all other substances

## Discussion

This retrospective chart review provides a descriptive assessment of health outcomes and healthcare utilization among Indigenous men who entered a MAP at its opening in December 2020. The program was delivered within an urban Indigenous-led community organization and integrated on-site addiction medicine services to address severe alcohol use and homelessness through a harm reduction approach. Outcomes were assessed for the one-year period prior to program initiation (December 2019 to December 2020) and during follow-up for up to one year following enrollment (through December 2021), with follow-up duration varying across participants. This study offers a complementary quantitative description of health service engagement and substance use outcomes associated with the MAP, alongside previously reported qualitative findings [19]. During the observed follow-up period, residents demonstrated increased engagement with health services, including follow-up care with medical specialists, improved access to prescription medications for underlying health conditions, and a reduction in emergency department utilization (Tables 2 and 3). Concurrently, reductions in alcohol consumption and use of other substances, including opioids, stimulants, and tobacco, were also observed (Tables 3 and 4).

This integrated model represents a preliminary example of Indigenous harm reduction within MAP settings and highlights an important direction for Indigenous-led harm reduction models aimed at enhancing access to health services for Indigenous Peoples experiencing homelessness and severe alcohol use. Although the present evaluation focused on Indigenous men, similar models may benefit other Indigenous populations and contribute to the growing literature on Indigenous-led harm reduction.

Indigenous-led harm reduction strategies are increasingly recognized in Canada; however, published evaluations remain limited, particularly for MAPs delivered within Indigenous-governed organizations or explicitly grounded in culturally safe care. Accounts from Indigenous participants in western harm reduction programs have emphasized the importance of integrating cultural supports alongside harm reduction approaches in Indigenous contexts [10]. Indigenous harm reduction has been described as centering Indigenous knowledge systems, cultural values, and community-defined priorities in responses to substance use [22]. These principles are reflected in culturally supportive housing initiatives, such as Ambrose Place, which emphasize cultural safety and community connection when supporting individuals experiencing homelessness and substance use [23].

The harm reduction approach implemented within the MAP and upheld by the CO reflects key elements of this framework. While maintaining core harm reduction principles such as meeting individuals where they’re at, staff and medical team members engaged residents through practices that emphasized learning, acceptance, and opportunities for cultural connection, where appropriate to each individual. This approach recognized the diversity of Indigenous identities and life experiences among residents. Similarly, a feasibility study examining the integration of an Indigenous-led MAP in British Columbia identified access to Indigenous cultural supports and community connection as key culturally grounded components of effective program design [24]. Together, these findings suggest that integrating cultural considerations within harm reduction programming may support more contextually appropriate and acceptable care for Indigenous Peoples. Recent literature further underscores that Indigenous harm reduction is most effective when grounded in holistic, community-driven approaches that integrate cultural practices and Indigenous healing frameworks, while addressing concerns related to the cultural appropriateness of services [25].

Within the present study, the MAP was embedded in an urban Indigenous-led community organization and incorporated several elements consistent with these approaches, including a culturally safe, non-abstinence-based environment; respect for residents’ cultural identities and lived experiences; and integration with supportive health services. Within this context, residents were able to engage with healthcare while achieving relative stabilization of alcohol consumption.

Although the proportion of residents hospitalized did not change over time, hospital admissions should not necessarily be interpreted as negative outcomes. Rather, they may reflect improved access to healthcare and more appropriate medical management compared with the treatment gaps often experienced by individuals living in street-based environments. However, this interpretation should be made cautiously, given variation in program participation duration. Prior research has documented that individuals entering MAPs often experience frequent cycling through emergency services with unmet health needs prior to enrollment [10]. Persistent mistrust of healthcare systems among people who use substances further highlights the importance of harm reduction models which have been shown to reduce stigma and foster engagement in care, particularly when grounded in principles of cultural safety [25].

A reduction in ED utilization, alongside sustained engagement with routine medical care, may suggest that participants’ healthcare needs have been addressed more comprehensively than alcohol use alone. These findings are consistent with existing evidence indicating that MAPs and other “wet” services reduce ED utilization, by up to 24%, through consistent access to medical professionals [9]. Moreover, MAP participants are more than twice as likely to report regular access to care, and three times more likely to report acceptable access, compared to periods without MAP participation [26]. The reduction in ED use observed in this study may be further attributable, in part, to the integration of clinical services within a culturally safe housing environment; an approach that is particularly relevant given that homelessness, Indigenous identity substance use, and mental illness are each independently associated with high ED utilization [27].

The observed decrease in daily alcohol consumption also warrants further consideration. As previously reported in the qualitative findings from this MAP, alcohol use for many residents was closely tied to survival-based drinking [19]. An Indigenous-led alcohol harm reduction program in Canada demonstrated how Indigenous-led environments may support meaningful shifts in individuals’ relationships with alcohol, moving from survival-driven use toward greater choice, control, and stability [28]. Such patterns are often rooted in displacement resulting from colonization and relocation to urban settings, where alcohol may function not only as a coping mechanism, but also as a source of social and community connection [10, 29]. Within MAPs, the structured and regulated provision of alcohol may therefore support reductions in harmful alcohol use while allowing residents to maintain social and community connections that were previously central to survival in street-based contexts [30].

### Future directions

Further research is needed to better understand the impact of Indigenous-led MAPs that integrate clinical care within culturally safe, community-based approaches to severe alcohol use. Emerging harm reduction models highlight the importance of Indigenous-led alcohol harm reduction models that extend beyond Western biomedical paradigms, particularly in light of the limitations of detoxification and abstinence-based services, which have failed to adequately account for Indigenous cultural, historical and social contexts [28]. Future directions should include the expansion of Indigenous-led MAPs to additional communities in Montréal, as well as to non-urban and remotely located settings, where access to addiction medicine services remains limited. Program development should also prioritize tailored models for specific populations, including young Indigenous adults as a preventative strategy, as well as for Indigenous women and Two-Spirit people, whose experiences and needs have historically been underrepresented in MAP programming and evaluation. Finally, further research is warranted to examine the potential role of MAPs with enhanced or specialized medical services for individuals with complex health needs, including those experiencing cognitive impairment, neurocognitive disorders, or dementia. Such adaptations may be critical to ensuring that MAPs remain responsive, inclusive, and capable of addressing the evolving health profiles of Indigenous Peoples experiencing homelessness and severe alcohol use.

### Limitations

Several limitations should be considered when interpreting these findings. First, this review is limited to outcomes among Indigenous men, reflecting the population initially served by the program. Although the MAP has since expanded to include Indigenous women and Two-Spirit individuals, their experiences were not captured in this evaluation and warrant further study. The term *Two-Spirit* encompasses diverse identities and meanings that are shaped by the individuals who carry the identity, their Nations, communities, and cultural contexts, rather than reflecting a singular universal definition [31].

Second, outcomes were derived from a small and diverse pool of First Nations and Inuit men experiencing homelessness and severe alcohol use. This reflects the program’s residential capacity at a single point in time and limits the generalizability of findings. Follow-up duration varied across participants, as residents entered and exited the program at different times, including due to involuntary discharge, death, or suspension. As a result, outcomes assessed at up to one year following program initiation may not fully capture longer-term impacts.

Third, this evaluation was descriptive in nature, and causal relationships between program participation and observed outcomes cannot be established. This limitation is compounded by incomplete access to healthcare utilization data, as residents often received care from multiple institutions and centralized hospital records across Montreal were not available, potentially resulting in underestimation of service use.

Fourth, the urban setting of the program represents an additional consideration. While all residents were living in Montréal prior to MAP enrollment, distance from home communities may influence opportunities for cultural connection and support. Access to community-specific cultural practices, Elders, and relationships may differ in urban environments compared with home communities. At the same time, the program’s proximity to a university hospital and access to integrated clinical services may limit transferability to other settings, including non-urban and remote communities. However, this integration is also a defining feature of the model and may inform adaptations in other contexts.

Finally, documentation related to the harmful consequences of alcohol use was frequently incomplete during follow-up, representing a notable limitation. As a result, no firm conclusions can be drawn regarding changes in substance-related harms, and more systematic evaluation of these outcomes is warranted in future studies.

## Conclusion

An integrated model of care within a community-based managed alcohol program offers a promising approach for Indigenous Peoples experiencing homelessness and severe alcohol use in urban settings. By combining Western biomedical care with culturally safe housing and services, alongside regulated alcohol provision, MAPs may support alcohol stabilization, management of complex health conditions, and engagement with healthcare beyond emergency services. This study provides preliminarily evidence that partnerships between Indigenous-led community organizations and integrated addiction medicine teams can generate culturally grounded, sustainable harm-reduction and health solutions. To the best of our knowledge, this is among the first evaluations of a MAP documenting such an integrated Indigenous-led clinical collaboration in Canada. These findings underscore the relevance of this model for harm-reduction practice and highlight the need for continued development, evaluation, and adaptation of similar approaches across diverse Indigenous and community contexts.

## Data Availability

Due to the nature of this research, the participants did not consent to have their data shared publicly; therefore, supporting data is not available.

## Abbreviations

MAP: Managed alcohol program
UH: University Hospital
ED: Emergency department
AUD: Alcohol use disorder
CO: Community organization
SUD: Substance use disorder
COVID-19: Coronavirus disease 2019
DSM-5: Diagnostic and statistical manual of mental disorders, Fifth Edition
HIV: Human immunodeficiency virus
PTSD: Post traumatic stress disorder
COPD: Chronic obstructive pulmonary disease
GHB: Gamma-hydroxybutyrate

## References

[1] Aubry T, Klodawsky F, Coulombe D. Comparing the housing trajectories of different classes within a diverse homeless population. Am J Community Psychol. 2012;49(1–2):142–55. 10.1007/s10464-011-9444-z 21557093

[2] Ministère de la Santé et des Services sociaux. Dénombrement des personnes en situation d’itinérance visible au Québec: rapport de l’exercice du 11 octobre 2022. Québec: Ministère de la Santé et des Services sociaux; 2023. https://publications.msss.gouv.qc.ca/msss/document-003630/.

[3] Statistics Canada. Focus on Geography Series, 2021 Census—Montréal (Census metropolitan area). Ottawa: Statistics Canada; 2022. https://www12.statcan.gc.ca/census-recensement/2021/as-sa/fogs-spg/Page.cfm?lang=E&topic=8&dguid=2021S0503462.

[4] Reading CL, Wien F. Health inequalities and the social determinants of Aboriginal peoples’ health. Prince George: National Collaborating Centre for Aboriginal Health; 2009.

[5] Goodman A, Fleming K, Markwick N, Morrison T, Lagimodiere L, Kerr T. “They treated me like crap and I know it was because I was Native”: The healthcare experiences of Aboriginal peoples living in Vancouver’s inner city. Soc Sci Med. 2017;178:87–94. 10.1016/j.socscimed.2017.01.053 PMC5367883 28214449

[6] Graham S, Muir NM, Formsma JW, Smylie J. First Nations, Inuit and Métis peoples living in urban areas of Canada and their access to healthcare: a systematic review. Int J Environ Res Public Health. 2023;20(11):5956. 10.3390/ijerph20115956 PMC10252616 37297560

[7] Nelson SE, Wilson K. Understanding barriers to health care access through cultural safety and ethical space: Indigenous people’s experiences in Prince George, Canada. Soc Sci Med. 2018;218:21–7. 10.1016/j.socscimed.2018.09.017 30316132

[8] Zhao J, Stockwell T, Pauly B, Wettlaufer A, Chow C. Participation in Canadian managed alcohol programs and associated probabilities of emergency room presentation, hospitalization and death: a retrospective cohort study. Alcohol Alcohol. 2022;57(2):246–60. 10.1093/alcalc/agab078 34999760

[9] Flores-Aranda J, Toussaint P. Revue systématique sur les modalités et les effets de services de type wet. Montréal: Institut universitaire sur les dépendances du CIUSSS du Centre-Sud-de-l’Île-de-Montréal; 2018.

[10] Pauly B, Gray E, Perkin K, Chow C, Vallance K, Krysowaty B, et al. Finding safety: a pilot study of managed alcohol program participants’ perceptions of housing and quality of life. Harm Reduct J. 2016;13:1–11. 10.1186/s12954-016-0102-5 PMC4860766 27156564

[11] Pauly B, Brown M, Evans J, Gray E, Schiff R, Ivsins A, et al. “There is a place”: impacts of managed alcohol programs for people experiencing severe alcohol dependence and homelessness. Harm Reduct J. 2019;16:1–14. 10.1186/s12954-019-0332-4 PMC6916004 31842903

[12] Pauly BM, Stockwell T, Chow C, Gray E, Krysowaty B, Vallance K, et al. Towards alcohol harm reduction: preliminary results from an evaluation of a Canadian managed alcohol program. Victoria: Centre for Addictions Research of British Columbia; 2013.

[13] Reed M. Wet shelters: the benefits and risks associated with alcohol-administering homeless shelters—a scoping review. Victoria: Knowledge and Information Services, Office of the Chief Information Officer, Ministry of Labour and Citizens’ Services, Government of British Columbia; 2008.

[14] Vallance K, Stockwell T, Pauly B, Chow C, Gray E, Krysowaty B, et al. Do managed alcohol programs change patterns of alcohol consumption and reduce related harm? A pilot study. Harm Reduct J. 2016;13:1–11. 10.1186/s12954-016-0103-4 PMC4860767 27156792

[15] Stockwell T, Pauly B, Chow C, Erickson RA, Krysowaty B, Roemer A, et al. Does managing the consumption of people with severe alcohol dependence reduce harm? A comparison of participants in six Canadian managed alcohol programs with locally recruited controls. Drug Alcohol Rev. 2018;37(S1):S159–66. 10.1111/dar.12618 29027283

[16] Nolan S, Fairgrieve C, Dong H, Garrod E, van Heukelom H, Parappilly BP, et al. A hospital-based managed alcohol program in a Canadian setting. J Addict Med. 2023;17(2):190–6. 10.1097/ADM.0000000000001080 PMC10062701 36149000

[17] American Psychiatric Association. Diagnostic and statistical manual of mental disorders. 5th ed. Washington: American Psychiatric Publishing; 2013.

[18] Prochaska JO, DiClemente CC. Stages and processes of self-change of smoking: toward an integrative model of change. J Consult Clin Psychol. 1983;51(3):390–5. 10.1037//0022-006x.51.3.390 6863699

[19] Motta-Ochoa R, Marsan S, Incio-Serra N, Audéoud ER, Talbot A, Mbacfou Temgoua M, et al. Barriers and facilitators to implementing a first managed alcohol program in Montreal, Canada. Drugs Educ Prev Policy. 2025;25:1–2.

[20] Canadian Institute for Substance Use Research. Standard drink calculator. Victoria: University of Victoria; 2025. http://aodtool.cfar.uvic.ca/en/index_stddt.html.

[21] Canadian Institutes of Health Research, Natural Sciences and Engineering Research Council of Canada, Social Sciences and Humanities Research Council of Canada. Tri-Council Policy Statement: Ethical Conduct for Research Involving Humans—TCPS 2 (2022). Ottawa: Secretariat on Responsible Conduct of Research; 2022. Chapter 9, Research Involving the First Nations, Inuit and Métis Peoples of Canada.

[22] Lavalley J, Kastor S, Valleriani J, McNeil R. Reconciliation and Canada’s overdose crisis: responding to the needs of Indigenous Peoples. CMAJ. 2018;190(50):E1466–7. 10.1503/cmaj.181093 PMC6291395 30559278

[23] Dumais N, Pascal N, Cunningham C, Hodgson M, Auger R, Dexter S, et al. tawāw pe-apik | poohsapoot, amo ihtopiit | edanigha, hoɁa | annaii t’sat dhiindii ts’at nizheh da’on tinich’uh | qain, aimaruatun aquviatin | come and sit and be at home. Victoria: Canadian Institute for Substance Use Research, University of Victoria; 2018.

[24] Child Y, McBeth Beausoleil R, Hunt-Jinnouchi F, Onespot-Whitney J, Brown M, Owens T. Indigenous pathways to health and wellbeing: Managed alcohol program feasibility study. Victoria: Aboriginal Coalition to End Homelessness Society; 2018.

[25] Zolopa C, Clifasefi SL, Dobischok S, Gala N, Fraser-Purdy H, Phillips MK, et al. A scoping review of harm reduction practices and possibilities among Indigenous populations in Australia, Canada, and the United States. Drug Alcohol Depend. 2025. 10.1016/j.drugalcdep.2025.112597. 39970577

[26] Davis A, Pauly B, Stewart SH, Stockwell T, Asbridge M. Access to healthcare among managed alcohol program participants: a mixed methods study. Int J Drug Policy. 2025;145:104950. 10.1016/j.drugpo.2025.104950 40829298

[27] Chambers C, Chiu S, Katic M, Kiss A, Redelmeier DA, Levinson W, et al. High utilizers of emergency health services in a population-based cohort of homeless adults. Am J Public Health. 2013;103(S2):S302–10. 10.2105/AJPH.2013.301397 PMC3969147 24148033

[28] Brown M, Hunt-Jinnouchi F, Robinson J, Clark N, Mushquash C, Milaney K, et al. “Give me the reins of taking care of myself with a home”: healing environments in an Indigenous-led alcohol harm reduction program. Harm Reduct J. 2024;21(1):177. 10.1186/s12954-024-01090-y PMC11426064 39327559

[29] Montreal Indigenous Community Network. Annual report 2023–2024. Montréal (QC): Montreal Indigenous Community Network; 2024. https://reseaumtlnetwork.com/wp-content/uploads/2024/11/Annual_report_23-24_NETWORK.pdf.

[30] Pauly B, King V, Smith A, Tranquilli-Doherty S, Wishart M, Vallance K, et al. Breaking the cycle of survival drinking: insights from a non-residential, peer-initiated and peer-run managed alcohol program. Drugs Educ Prev Policy. 2021;28(2):172–80.

[31] Hunt S. An introduction to the health of two-spirit people: historical, contemporary and emergent issues. Prince George: National Collaborating Centre for Aboriginal Health; 2016.

